# Bevacizumab side effects and adverse clinical complications in colorectal cancer patients: review article

**DOI:** 10.1097/MS9.0000000000000981

**Published:** 2023-06-26

**Authors:** Rahmawati Minhajat, Tutik Harjianti, Itzar Chaidir Islam, Sanjaya Winarta, Yason Nikolaus Liyadi, Nabilah Putri Bamatraf, Rabbaniyah Amanuddin

**Affiliations:** a Internal Medicine Department, Hematology and Medical Oncology Division; b Medical Education Department; c Medical Doctor Program, Faculty of Medicine, Hasanuddin University, Makassar, Indonesia

**Keywords:** adverse events, bevacizumab, colorectal cancer, quality of life

## Abstract

This review aimed to evaluate the effect of bevacizumab on adverse events and the quality of life (QoL) from colorectal cancer (CRC) patient.

**Methods:**

A literature search was conducted from PUBMED, MEDLINE databases and some valid literatures from another databases that published in the last 10 years, and the design is an observational and randomized controlled trial (RCT).

**Results:**

CRC is a malignancy, which happens in the colon or rectum. One of the therapy for CRC is by using bevacizumab, a monoclonal antibody. However, the usage of bevacizumab is still become a controversy because of its clinical complications. Several studies showed that bevacizumab could cause some adverse events in the cardiovascular, gastrointestinal, hematology, and others body systems and affect the CRC patient QoL. However, the clinical complication of this drug is also affected by the combination therapy regimen used.

**Conclusions:**

The use of bevacizumab could cause some adverse event in different aspects, including cardiovascular, gastrointestinal, hematology, and others. Some of those are significant, but others are not. Besides that, using bevacizumab as a treatment regiment could also affect the QoL of CRC patients, but it is also affected by the combination therapy regimen used.

## Introduction

Highlights

- Colorectal cancer (CRC) is the third most common and deadly malignancy.
- Most CRC with metastatis are handled with a combination of cytotoxic and biological agent.
- Current treatment normally includes a fluoropyrimidine-based doublet or fluoropyrimidine monotherapy.
- This review aimed to evaluate the effect of bevacizumab on adverse events and the quality of life from CRC patients.

Colorectal Cancer (CRC) is the third most common malignancy and the second most deadly cancer, which was estimated around 1.93 million cases and 0.94 million deaths in 2020 worldwide, representing 10% of the global cancer incidence (total 10.20 new cases) and 9.4% of all cancers that caused deaths (total 9.96 million deaths). CRC is the third leading cause of cancer related deaths in both sexes worldwide, with estimated 515 637 deaths among males and 419 536 deaths among females in 2020^1^. The lack of genomic and/or epigenomic stability has been found within the majority of early neoplastic lesions in the colon and is probably a primary molecular and pathophysiological event in the initiation and formation of CRC^2^. The preference for first-line treatment for CRC patients presently includes a multimodal method based on tumor-associated characteristics (e.g. wide variety and localization of metastases, tumor progression, presence or absence of biochemical markers, etc.) and patient-associated factors (e.g. co-morbidity, prognosis, etc.). In practice, some of these factors are used to categorize CRC patients into four different risk groups to be used to guide the treatment strategy^3^. Most CRC with metastatis are handled with a combination of cytotoxic and biological agent. Current treatment normally includes a fluoropyrimidine-based doublet (FOLFOX/CAPOX or FOLFIRI/CAPIRI) or fluoropyrimidine monotherapy (5-FU/folinic acid or capecitabine) mixed with a biological agent focused on either the vascular endothelial growth factor (VEGF) in an unselected populace or the epidermal growth factor receptor in patients with RAS wild-type tumor^4^. This systematic overview focuses on bevacizumab, the agent’s most commonly used VEGF-directed monoclonal antibody, which was accepted by the United States Food and Drug Administration and is available in the marketplace based on its effectiveness in metastatic cancers. Bevacizumab particularly binds to the VEGF-A protein, thereby inhibiting the mechanism of angiogenesis. However, there is some debate about bevacizumab adverse drug reactions, with some studies finding increased risks of the occurrence of some adverse drug reactions, such as all-grade hypertension (risk ratio (RR): 7.5, 95% CI: 4.2–13.4 vs. RR: 3.0, 95% CI: 2.2–4.2) and high-grade bleeding (RR: 3.02, 95% CI: 1.87, 95% CI: 0.95–1.7) for the high-dose^5^. It is anticipated that this effect may have different impacts on a patient’s quality of life (QoL). Based on these findings, the purpose of this paper was to review the results of studies that analyzed the adverse events and QoL by adding bevacizumab to patients with CRC treatment.

## Methods

### Literature search

In the preparation of this systematic review, a literature search was conducted from PUBMED and MEDLINE databases with the keywords used are (((colorectal cancer) AND (bevacizumab)) AND ((cardiovascular) OR (intestinal perforation))). Furthermore, some valid literatures from another databases were added to this systematic review if meet the selection criteria of this study.

### Study eligibility and screening criteria

The study criteria that will be included in this systematic review are: Publication in the last 10 years; The design is an observational and randomized controlled trial (RCT); The language used is Indonesia or English; One of the exposures is bevacizumab; and The outcomes are adverse event and QoL. Meanwhile, the study criteria that will be excluded are: No abstract; Unavailable access for full text; and Incorrect article type.

Literature studies that match the eligibility criteria will be included while those that do not match the criteria will be excluded with further explanation. The conflicts in grouping the studies will be discussed together until a conclusion is reached. The screening result will later be reported using the Preferred Reporting Items for Systematic Reviews and Meta-analyses (PRISMA) guidelines.

### Data collection

Data collection will be carried out for all included studies. The data that will be collected include: The main author; The year of publication; The place where the research was conducted; The study design; The number of samples; The mean/range age of samples; Type of Exposure; Type of Outcome.

Data collection was carried out by four reviewers (S.W., R., N.P.B., and Y.N.L.) and then cross-checked by other reviewers (R.M., I.C.I., T.H.). If in the included literature study there is incomplete data, the reviewer will contact the author of the study, if the author does not respond, the study is then excluded with the reviewer agreement.

### Quality assessment

Quality assessment in this systematic review was carried out by six reviewers ( R.M., I.C.I., S.W., R., N.B.P., and Y.N.L.). using the Newcastle–Ottawa Scale for observational study. In a study with a cross sectional and case–control design, an assessment was carried out on three main aspects, selection, comparability, and exposure. Meanwhile, in a study with a cohort design, the exposure aspect was replaced by assessing the outcome aspect. The assessment is done by discussing point by point, every point that has a low risk of bias we will give one star, except for the aspect of compatibility which allows for two stars. Studies with more stars show a better quality of study.

The RCT was assessed using the Cochrane risk of bias tools by assessing seven domains. Random Sequence Generation; Allocation concealment; Blinding of participants and personnel; Blinding of outcome assessment; Incomplete outcome data; Selective reporting; and Other bias. Each domain will be given an assessment of low risk, high risk, and unclear. The results of quality assessments from RCT studies are then reported in graphical form. The work has been reported in line with AMSTAR (Assessing the methodological quality of systematic reviews) guidelines.

## Results

### Literature search and screening

**Figure 1:**
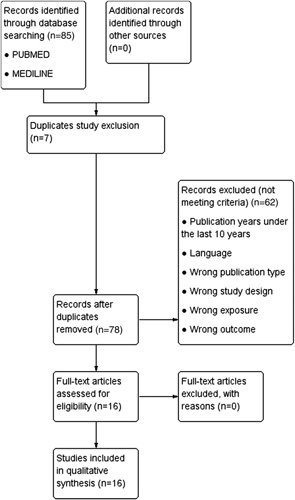
Preferred reporting items for systematic reviews.

We found 85 studies after searching from PUBMED and MEDLINE database using the keywords. All of these studies were screened by four reviewers (S.W., R., N.P.B., and Y.N.L.). A total of 69 studies were excluded due to duplicate study, publication years under the last 10 years, inappropriate language, wrong publication type, study design, exposure, and outcome leaving 16 studies that were included in the qualitative synthesis. Figure 1 depicts the entire set of search and filter results.

### Characteristics of the eligible study

There are four clinical trial, seven case–control, and five cohort studies in this systematic review, which were done in 14 different countries with the total sample size were 21.674 people. Furthermore, those studies assessed the adverse event and the QoL of CRC patients using bevacizumab, in which nine studies assessed the cardiovascular, eight studies assessed the gastrointestinal or hematological or other adverse events, and two studies assessed the QoL from CRC patients using bevacizumab. Characteristics of the eligible studies were summarized in Table 1.

**Table 1:** Characteristics of inclusion studies

No	References	Country	Study design	Sample size	Mean Age/ Range	Exposure	Outcome
1	Kapelakis^6^	Greece	Cohort study	35	64.81	Bevacoxaliplatin (130 mg/m²) + capecitabine (2000 mg/m² ) with or without bevacizumab (15 mg/kg).	Cardiovascular adverse event (coronary artery disease, myocardial infarction, thromboembolic event).
2	Ina^19^	Japan	Case–control	130	41–85	Bevacizumab + oxaliplatin, 5-fluorouracil + leucovorin (modified FOLFOX-6) + capecitabine + oxaliplatin + irinotecan + 5-fluorouracil + leucovorin (FOLFIRI) + S1 plus irinotecan (IRIS), or a bolus injection of 5-fluorouracil (400 mg/m²) on day 1 and continuous infusion of 5-fluorouracil (2400 mg/m²) over 46 hours plus l-leucovorin (200 mg/m²).	Cardiovascular adverse event (thrombosis), hematology adverse event (hemorrhage), other adverse event (interstitial penumonitis, tuberculosis).
3	Bennouna^11^	Germany	Clinical trial	810	63 (27–84)	Infusional or bolus fluorouracil or oral capecitabine at the investigator’s discretion + irinotecan or oxaliplatin with or without bevacizumab at 2.5 mg/kg per week equivalent (5 mg/kg intravenously every 2 weeks or 7.5 mg/kg intravenously every 3 weeks).	Cardiovascular adverse event (venous thromboembolism), gastrointestinal adverse event (gastrointestinal perforation), hematology adverse event (bleeding, neutropenia), other adverse event (asthenia).
4	Avallone^18^	Italy	Clinical trial	230	18–75	12 biweekly cycles of a modified FOLFOX-6 regimen (intravenous oxaliplatin, 85 mg/m^2^ , on day 1, followed by intravenous levo–folinic acid, 200 mg/m^2^ + bolus fluorouracil, 400 mg/m^2^ + 46 h intravenous administration of fluorouracil, 2400 mg/m^2^ ) or a modified CAPOX regimen (intravenous oxaliplatin, 85 mg/m^2^ , on day 1 + oral capecitabine, 1000 mg/m^2^ , twice daily on days 1 to 10) every 2 weeks for 12 cycles. Bevacizumab (5 mg/kg) was administered on the same day as chemotherapy (standard arm) or 4 days before (experimental arm).	Gastrointestinal adverse event (diarrhea, nausea, abdominal pain), other adverse event (proteinuria), quality of life.
5	Baek^16^	Korea	Case–control	488	59 (36–68)	Bevacizumab (5 mg/kg) + FOLFIRI (130-180 mg/m^2^) + 5-Fluoroacil (400 mg/m^2^) + Leucovorin (20 mg/m^2^).	Gastrointestinal adverse event (gastrointestinal perforation).
6	Cao^20^	China	Case–control	71	54 (25–75)	5-fluorouracil/leucovorin-based chemotherapy +bevacizumab. Bevacizumab 5 mg/kg was administered every 2 weeks according to 5-FU-based chemotherapy regimens (FOLFIRI or FOLFOX) or 7.5 mg/kg every 3 weeks according to a capecitabine-based regimen (XELOX).	Hematology adverse event (bleeding).
7	Reglat	France	Cohort study	1550	65.1 (21.9–84.3)	FOLFIRI regimen associated with bevacizumab. Mean (SD) starting bevacizumab dose was 5.1 (0.5) mg/kg.	Cardiovascular adverse event (hypertension, thrombotic events), gastrointestinal adverse event (gastrointestinal perforation, nausea, vomiting, diarrhea), hematology adverse event (bleeding, epistaxis, neutropenia, anemia, thrombocytopenia), other adverse event (proteinuria, asthenia).
8	Căinap	Roma	Case–control	151	57 (19–75)	Single-dose bevacizumab (5 mg/kg every 2 weeks or 7.5 mg/kg every 3 weeks) or double dose bevacizumab (10 mg/kg/every 2 weeks or 15 mg/kg every 3 weeks).	Cardiovascular adverse event (hypertension, thromboembolic event), other adverse event (proteinuria).
9	Tsai^7^	United states	Cohort study	6083	≥65	Regimen cycle BEV, 5-FU, capecitabine, oxaliplatin, irinotecan, and epidermal growth factor receptor inhibition.	Cardiovascular adverse event (arterial thromboembolic event, cardiac death, cardiomyopathy, congestive heart failure).
10	Gruenberger^17^	United kingdom	Clinical trial	80	32–77	Bevacizumab (5 mg/kg) + mFOLFOX- 6 [oxaliplatin 85 mg/m^2^, folinic acid 400 mg/m^2^, 5-fluorouracil 400 mg/m^2^ (bolus) then 2400 mg/m^2^ (46 h infusion)] or FOLFOXIRI [oxaliplatin 85 mg/m^2^, irinotecan 165 mg/m^2^, folinic acid 200 mg/m^2^, 5-fluorouracil 3200 mg/m^2^ (46 h infusion)] every 2 weeks.	Gastrointestinal adverse event (diarrhea), hematology adverse event (neutropenia, febrile neutropenia).
11	Koca^13^	Turkey	Case–control	172	18–81	Oxiplatine based regime and mFOLFIRI-B regime (folinic acid 400 mg/m^2^ + 5-FU 400 mg/m^2^ bolus + 5-FU 2.400 mg/m^2^ 46 h infusion + irinotecan 180 mg/m^2^ + bevacizumab 5 mg/kg every 14 days).	Cardiovascular adverse event (hypertension), hematology adverse event (neutropenia), other adverse event (proteinuria).
12	Yamazaki^21^	Japan	Clinical trial	395	26–75	Bevacizumab (5 mg/kg) followed by FOLFIRI (irinotecan 150 mg/m^2^; l-leucovorin 200 mg/m^2^; intravenous bolus of fluorouracil 400 mg/m^2^, continuous infusion of fluorouracil 2400 mg/m^2^), or bevacizumab followed by mFOLFOX6 (oxaliplatin 85 mg/m^2^ instead of irinotecan).	Quality of life
13	Kabbinavar^14^	United States	Cohort study	1953	–	Bevacizumab	Gastrointestinal adverse event (gastrointestinal perforation).
14	Vallerio^8^	Italy	Cohort study	7747	–	Bevacizumab	Cardiovascular adverse event (thrombotic events, pulmonary embolism, heart failure, rhythm disorder).
15	Bencsikova^9^	Czech Republic	Case–control	1622	22–85	The chemotherapy regimens were as follows: FOLFOX4 (oxaliplatin 85 mg/m^2^ IV day 1; leucovorin 200 mg/m^2^ IV days 1 and 2; 5-FU bolus 400 mg/m^2^ IV days 1 and 2; 5-FU 600 mg/m^2^ IV 22 h continuous infusion days 1 and 2 every 2 weeks), FOLFIRI (irinotecan 180 mg/m^2^ IV day 1; leucovorin 200 mg/m^2^ IV day 1 and 2; 5-FU 600 mg/m^2^ IV 22 h continuous infusion days 1 and 2 every 2 weeks), XELOX (oxaliplatin 130 mg/m^2^ IV day 1; capecitabine 1000 mg/m^2^ twice daily PO for 14 days every 3 weeks), or XELIRI (irinotecan 250 mg/m^2^ IV day 1; capecitabine 1000 mg/m^2^ twice daily PO for 14 days every 3 weeks). Bevacizumab was administered at a dosage of 5 mg/kg IV every 2 weeks or 7.5 mg/kg IV every 3 weeks depending on the chemotherapy regimen. Dosage of bevacizumab was not reduced.	Cardiovascular adverse event (thromboembolic complication, hypertension), gastrointestinal adverse event (gastrointestinal perforation), hematology adverse event (bleeding), other adverse event (proteinuria).
16	Bang^15^	Korea	Case–control	157	–	Bevacizumab 7.5 mg/kg on day 1 and capecitabine 1250 mg/m^2^ orally (PO) twice daily on day 1 to 14, was repeated every 3 weeks.	Gastrointestinal adverse event (gastrointestinal perforation), hematology adverse event (bleeding), other adverse event (hand-foot syndrome, proteinuria).

### Quality assesment

The quality of the 12 included observational studies was represented by the total number of stars obtained for each study ranging from 0 to 9. The lowest score was four stars two studies, five stars in five studies, six stars in three studies, and the highest score was eight stars in two studies. Table 2 contains the exact data from the quality assessment.

**Table 2:** Quality assessment result of the observational study using Newcastle–Ottawa scale

	S				C	E/O			
References	1	2	3	4	1	1	2	3	SUM
Kapelakis^6^	–	*	*	*	**	–	*	–	5
Ina^19^	*	*	–	*	**	*	*	*	8
Baek^16^	*	*	–	–	–	*	*	*	5
Cao^20^	*	*	–	–	–	*	*	*	5
Reglat	*	–	*	*	–	*	*	–	5
Căinap	*	*	*	*	–	*	*	–	6
Tsai^7^	*	*	*	*	**	*	*	–	8
Koca^13^	*	*	–	–	–	*	*	–	4
Kabbinavar^14^	*	*	*	*	–	*	*	–	6
Vallerio^8^	*	–	*	*	–	*	*	–	5
Bencsikova^9^	*	*	–	–	–	*	*	–	4
Bang^15^	*	*	*	*	–	*	*	–	6

**Figure 2:**
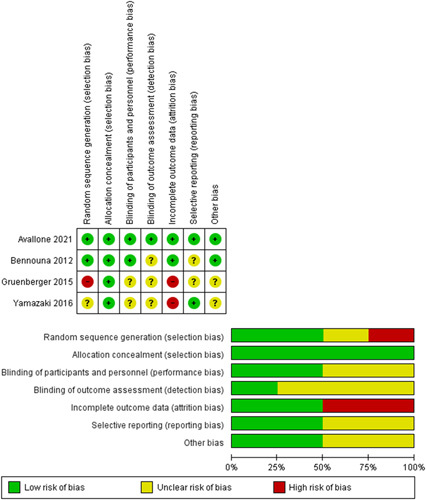
Quality assessment result of the experimental study using Cochrane risk of bias tools.

The quality of four clinical trials was assessed using graphs and a risk of bias summary. In general, those four studies have a good quality assessment, in which one study has a low risk of bias over the whole domain. Figure 2 displays the complete breadth of the quality evaluation.

## Discussion

Sixteen studies in this systematic review assess the adverse event and QoL from CRC patient using bevacizumab. The adverse events are divided into four categories, cardiovascular, gastrointestinal, hematology, and other. Bevacizumab has some adverse event on the cardiovascular system. Two of them are coronary artery disease and myocardial infarction in which the exact mechanism is still unknown^6^. Heart failure is the latest complication that could happen because of an adverse cardiovascular event. However, based on study conducted by Tsai and Valerio, heart failure was minimal in CRC patient receiving bevacizumab. Other cardiovascular adverse events that could happen are arrhythmia, cardiac death, and cardiamyopathy. The study by Vallerio reported that the type of arrhythmia that could happen are atrial fibrilation and atrioventricular block because of the acute hemodynamic effect by bevacizumab. Meanwhile, a study by Tsai and others also reported that cardiac death and cardiomyopathy were insignificant.

The thromboembolic event is also included as a cardiovascular adverse event on bevacizumab usage. The study by Bencsikova and Vallerio reported that thromboembolic event often happen in CRC patients receiving bevacizumab. On the other hand, studies conducted by Căinap, Bennouna, and Tsai reported that the thromboembolic event was minimal^8,13,14,19,20^. Meanwhile, a study by Kapelakis showed that people with past thromboembolic event belong to a high risk group of taking bevacizumab, so it should be administered carefully^6^.

The last cardiovascular adverse event that could happen is hypertension, which, some studies in this systematic review reported that hypertension was a significant side effect caused by bevacizumab toxicity^12,13,16,20^. On the other hand, a gastrointestinal adverse event caused by bevacizumab is GIT perforation^8,20^. Most GIT perforation occur within 6 months of starting bevacizumab therapy^8^. GIT perforation is known as bevacizumab’s effect^12,18^. The severe event was rare^20,21^ ,but was deemed as lethal^10^. One case of death was found with two intestinal perforation developed within 3 months^10^ Another study also reports a case of sepsis death due to perforation at the intestinal stenting procedure^5^. Bevacizumab lead to GIT perforation possibly by leading to necrosis of the tumor thus putting the patient at risk^10^. Another way might be limiting blood perfusion to the bowel area, that could lead to infarction and then perforation^10,18^. Diarrhea was also one of frequently adverse event reported^8,12,15^. One study found that registering bevacizumab on the same day as chemotherapy instead on registering it four days before was significantly associated with more diarrhea, nausea, and abdominal pain^9^.

In the hematological adverse event, the incidence of bleeding increased by adding bevacizumab to chemotherapy, but it was not statistically significant^7,8,11,12,20,21^. Some of the bleeding were clinically significant^7,8^ but some were not^11,12,20,21^. Bleeding might be the effect of the anti-VEGF of bevacizumab, inhibiting coagulation and endothelial proliferation^7^. Another most common adverse events reported in bevacizumab intervention was neutropenia^8,12,16^ and the most frequent was grades 3–4^15^. In these studies, neutropenia^8,15^ and febrile neutropenia^17^ were common in grades 3–5 (severe) adverse events. One case reported neutropenia as a treatment-related that result in death^8^. The other hematological events were anemia and thrombocytopenia, but the study did not stated those as statistically significant^12^. Other adverse events such as proteinuria was observed in the group with bevacizumab added to their therapy^9,12,13,16,20,21^. Proteinuria adverse event was statistically significant in one study^9^. Another study comparing double and single-dose bevacizumab administration found that grade-3 proteinuria reached statistical significance^13^. Asthenia was also a common severe adverse events, but studies did not stated it as statistically significant, considering asthenia was also common in the control group^8,12^. A study reported two cases of intersitial pneumonitis from bevacizumab group. It was not statistically significant, but this case raised concern because no pulmonary effect was found when the study reinitiated chemotherapy irinotecan without bevacizumab. However, this study did not found any differences in tuberculosis cases for both bevacizumab and control groups^7^. A case–control study reported hand-foot syndrome in 58,6% cases, following co-treatment with bevacizumab and capecitabine, in which grade-3 level occurred in 5% of patients^21^.

Meanwhile, from a QoL aspect, the study conducted by Avallone suggests that patients who were administered bevacizumab 4 days before chemotherapy (experimental group) have higher QoL scores at weeks 12 and 24 than patients who got bevacizumab at the same day with chemotherapy (control group), but the difference was not significant. The experimental group reported a significant increase in QoL regarding physical functioning and constipation score. More patients could cause this higher QoL to finish their treatment and maintenance therapy, and fewer patients get dose reduction in the experimental group^9^. In another study comparing FOLFIRI (irinotecan), bevacizumab, and mFOLFOX6 (oxaliplatin), bevacizumab showed that the sensory neurotoxicity effect lowers the QoL in 9 months after oxaliplatin and bevacizumab registered. This toxicity persists after the termination of oxaliplatin^17^.

## Conclusion

The use of bevacizumab could cause some adverse event in different aspects, including cardiovascular, gastrointestinal, hematology, and others. Some of those are significant, but others are not. Besides that, using bevacizumab as a treatment regiment could also affect the QoL of CRC patients, but it is also affected by the combination therapy regimen used.

## Ethical approval

The Ethics Committee of Hasanuddin University waived the need for ethics approval for this non-interventional study (review article). There are no specific permissions were required for corresponding locations due to the institutional regulation.

## Consent

Consent for review article does not necessarily.

## Sources of funding

No Financial support, funding, and sponsorship in this review study.

## Author contribution

All authors of this paper have directly participated in the planning, execution, or analysis of this study. R.M., T.H., and I.C.: concepting the work and study design; R.M., I.C., and S.W.: data acquisition and literature searching; R.M., S.W., and Y.S.: drafting the manuscript; R.M., N.P.B.,T.H., and R.: reviewing and editing for intellectual content; All authors read and approved the final manuscript.

## Conflicts of interest disclosure

No conflicts of interest of all authors in this study.

## Research registration unique identifying number (UIN)

The review was not registered

A protocol was not prepared; and

There is no protocol amendment.

## Guarantor

No Guarantor in this study.

## Availability of data, code, and other materials

The data that support the findings of this study are available on request from the corresponding author.

## Provenance and peer review

Not commissioned, externally peer reviewed.
